# Extensive environmental contamination and prolonged severe acute respiratory coronavirus-2 (SARS CoV-2) viability in immunosuppressed recent heart transplant recipients with clinical and virologic benefit with remdesivir

**DOI:** 10.1017/ice.2021.89

**Published:** 2021-03-12

**Authors:** Irina Rajakumar, Debra L. Isaac, Nowell M. Fine, Brian Clarke, Linda P. Ward, Rebecca J. Malott, Kanti Pabbaraju, Kara Gill, Byron M. Berenger, Yi-Chan Lin, David H. Evans, John M. Conly

**Affiliations:** 1 Alberta Health Services, Calgary, Alberta, Canada; 2 University of Calgary, Calgary, Alberta, Canada; 3 University of Alberta, Edmonton, Alberta, Canada; 4 Alberta Public Health Laboratory, Alberta Precision Laboratories, Calgary, Alberta, Canada

*To the Editor—*Remdesivir is an antiviral medication that exhibits antiviral activity versus SARS-CoV-2,^1–3^ but in clinical trials, it has demonstrated conflicting results with respect to mortality in patients with severe coronavirus disease 2019 (COVID-19).^4,^^5^ The use of remdesivir in immunosuppressed patients, including the initial posttransplant period with its high degree of immunosuppression, has not been well studied.^6–8^ We examined the virologic and clinical responses to remdesivir in 2 recent cardiac transplant cases with SARS-CoV-2 infection.

## Methods

Nasopharyngeal (NP) swabs, saliva, and clinical and environmental samples were collected at regular intervals beginning shortly after admission. They were tested using molecular assays^9^ and quantitative culture (Supplementary Material online). Patients provided informed consent with the approval of the University of Calgary’s Health Research Ethics Board (no. 20-0444).

### Case descriptions

Case 1 was a 56-year-old woman with a history of dilated cardiomyopathy with end-stage heart failure, type-2 diabetes mellitus, hypothyroidism, osteoporosis, and anemia. This patient underwent an orthotopic heart transplant with antithymocyte globulin (ATG) induction and standard triple immunosuppressive therapy. The patient was discharged 30 days later with prednisone, tacrolimus, and mycophenolate mofetil (MMF), and standard prophylactic medications.

At 5 days after discharge, she was rehospitalized following community exposure to COVID-19. Nasal stuffiness and discharge, sneezing, fatigue, and cough developed on admission day 4 and a NP swab was positive for SARS-CoV-2. All symptoms except fatigue had resolved by day 12. However, dyspnea, cough, and hypoxia developed on day 15 and the chest radiograph revealed new bibasilar interstitial infiltrates. Corticosteroids and antimicrobials were initiated for presumptive COVID-19 pneumonitis and superimposed bacterial pneumonia, but on day 21 oxygen requirements increased significantly.

**Fig. 1.**
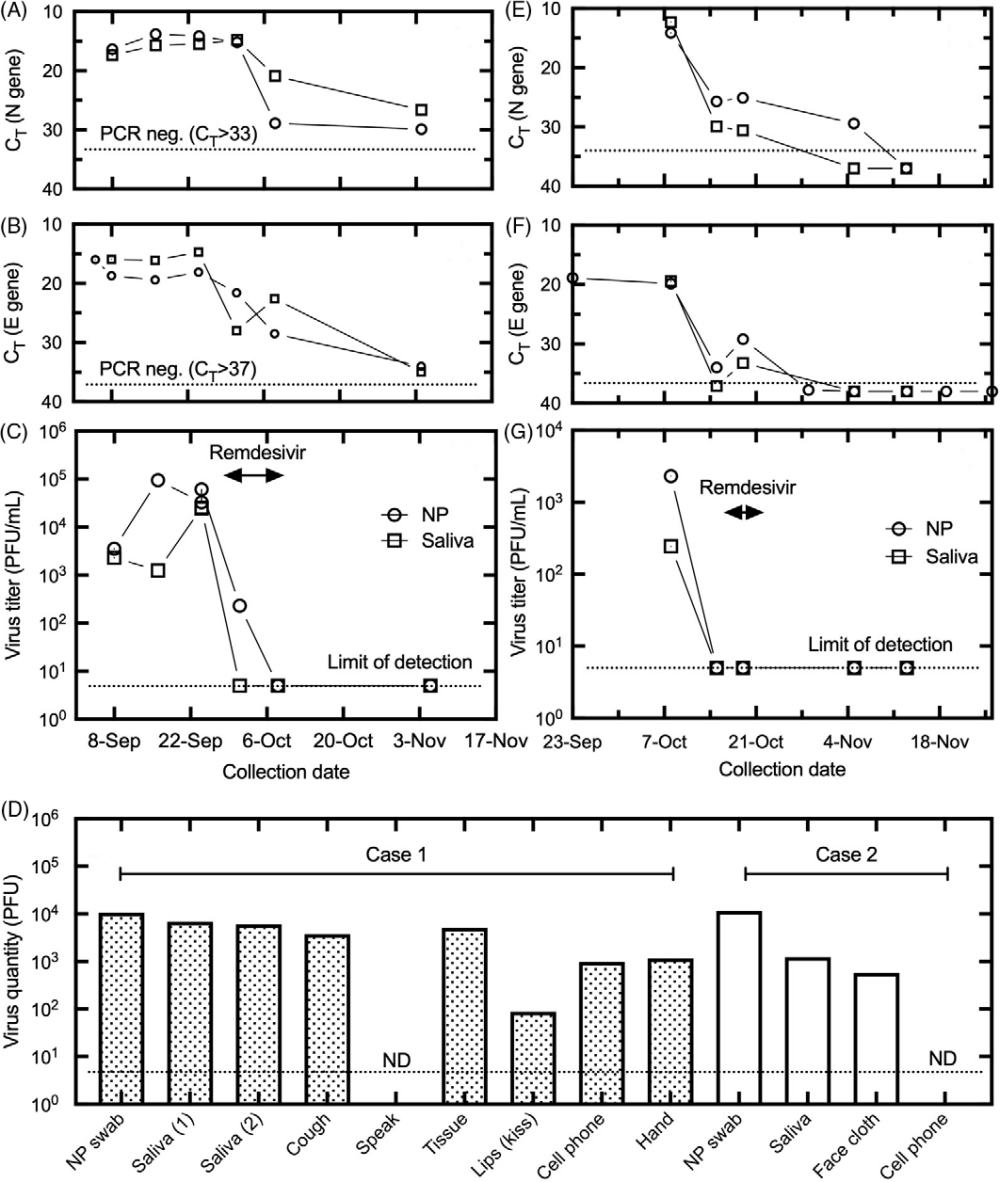
Results of nasopharyngeal (NP) and saliva specimens collected from cases 1 and 2 from E and N gene RT-PCR assays with cycle threshold (Ct) values (panels A and B plus E and F, respectively) and quantitative viral culture results on Vero cells (adjusted for volume) from case 1 and case 2 clinical samples (panels C and G, respectively). Culture results on Vero cells are shown for clinical and environmental samples for cases 1 and 2 (panel D). Of 6 environmental samples obtained at the bedside in case 1 (ie, kiss sample, discarded nasal tissues, cell phone, both hands, a cough bag and a 3–5-minute speaking bag), 5 had viable virus; the only exception was the speaking bag. Of 2 environmental samples obtained at the bedside in case 2 (a pledget cut from a face cloth found lying on the patient’s bed for at least 1 hour and the cell phone), the face cloth had viable virus whereas the cell phone did not. Dotted lines indicate the Ct value where no samples were positive and the limit of detection for PCR and culture, respectively. Note. PFU, plaque-forming units.

Despite discontinuation of MMF and reduction of tacrolimus, cultivatable viral loads increased in the nasopharynx and saliva (Fig. 1), the chest radiograph and clinical condition deteriorated, and mechanical ventilation was considered. Extensive contamination with high quantitative burdens of viable virus was detected in the patient’s immediate environment in the hospital room (Fig. 1). A 10-day course of remdesivir was initiated on day 27 (Fig. 1). The patient’s clinical condition and chest radiograph improved, allowing oxygen discontinuation by day 32 and discharge on day 44.

Case 2 was a 33-year-old woman with a history of end-stage heart failure secondary to congenital heart disease, liver cirrhosis, and kidney disease who underwent orthotopic heart transplant with antithymocyte globulin induction therapy. The patient was discharged after 4 weeks on prednisone, tacrolimus, MMF, and standard prophylactic medications.

This patient tested positive for SARS-CoV-2 shortly after discharge and was readmitted 1 week later with progressive dyspnea and hypoxemia requiring intubation. The MMF dose was reduced, and dexamethasone and antimicrobials were initiated. MMF was subsequently held but was later restarted when an echocardiogram demonstrated a reduction in left ventricular systolic function, suggesting acute graft rejection. Significant viable virus burdens were noted in the NP swab and saliva along with a face cloth found on the bed 16 days after initial SARS-CoV-2 positivity (Fig. 1). A 5-day course of remdesivir was initiated followed by clinical recovery and discharge 7 days later.

## Discussion

The first patient’s NP and salivary samples (Fig. 1) showed an increase in quantifiable SARS-CoV-2 in the context of the postcardiac transplant immunosuppressive regimen. The initial symptom resolution was not associated with a reduced viable viral load. Extensive contamination with replication- and infection-competent SARS-CoV-2 was detected ranging from 10^2^ to 10^4^ plaque-forming units (PFU) in a kiss sample, discarded nasal tissues, a cell phone, both hands, and cough specimens. The development of presumptive viral pneumonitis correlated with the highest levels of cultivatable virus, and MMF discontinuation had no impact on viral load. The initiation of remdesivir (Fig. 1) was associated with a dramatic decline in virus titers associated with a progressive clinical and radiologic improvement. The samples at day 10 of remdesivir treatment and 23 days after remdesivir revealed no cultivatable virus.

The second patient demonstrated a moderately high viral load (Fig. 1) 18 days after symptom onset. A face cloth found on the bed 16 days after initial SARS-CoV-2 positivity had ˜10^3^ PFU of replication– and infection–competent virus. Samples obtained immediately prior to remdesivir were E/N gene positive but were negative for virus recovery. However, the risk for viral reactivation was considered high given the reintroduction of MMF for early rejection. This patient clinically recovered with repeat specimens obtained at end-of-therapy and 25 days post-remdesivir revealing no cultivatable virus.

No published evidence demonstrates that remdesivir offers mortality or graft survival benefits to solid organ transplant (SOT) patients in the immediate posttransplant period. This scenario is likely too uncommon to permit adequately powered demonstration of utility in clinical trials. However, the need to reduce immunosuppressive therapy in SOT patients with COVID-19 creates a competing risk of acute rejection. The risk of graft loss and consequent mortality is substantial, and an agent that can reduce viral loads and permit restoration of immunosuppression can help mitigate that risk, especially in SOT recipients with COVID-19 who are within 6 months of transplantation or following a rejection episode.

These 2 cardiac transplant patients exhibited prolonged viable SARS-CoV-2 carriage from symptom onset, 35 and 26 days, respectively, with rising or unchanged viral loads despite decreasing doses of immunosuppressives in 1 case. Prolonged high viable viral carriage was also reported in hematology-oncology patients.^7,10^ Large quantities of viable SARS-CoV-2 may be shed in highly immunosuppressed patients for prolonged periods of time, creating major clinical and infection control challenges. Our findings of prolonged carriage with extensive environmental contamination adds support for the risk of SARS-CoV-2 infection via the disregarded role of direct contact/fomite transmission, highlighting the need to consider virological countermeasures, which may be a unique role for remdesivir.

## Supplementary material

For supplementary material accompanying this paper visit http://dx.doi.org/10.1017/ice.2021.89. click here to view supplementary material
