# Supplementary material for: Extensive environmental contamination and prolonged severe acute respiratory coronavirus-2 (SARS CoV-2) viability in immunosuppressed recent heart transplant recipients with clinical and virologic benefit with remdesivir
Source: Infect Control Hosp Epidemiol. 2021 Mar 12:1–3. doi: 10.1017/ice.2021.89 (PMC7985896; doi:10.1017/ice.2021.89)
Supplement: Supplementary file 1 [file icesup.zip › S0899823X21000891sup002.docx]

**Supplemental Material**

**Materials and Methods**

Each nasopharyngeal (NP) swab (Flexible Mini-tip FloqSwab, Copan) or saliva (~1 ml) specimen was collected into a sterile container to which was added 3 mL of Copan UTM-RT (Code 330C) or Dulbecco's Modified Eagle’s Medium (Gibco) with addition of 2% fetal bovine serum, 1 µg/ml meropenem and 1µg/ml Amphotericin B (DMEM +). Environmental samples were collected in a similar manner using either an NP swab (Flexible Mini-tip FloqSwab, Copan), a 2 x 2 cm gauze, or small pledgets cut from contaminated cloth samples pre-moistened with DMEM+, to which varying amounts of DMEM + were added, depending on the sample, trying to avoid overdilution. A single kiss sample (pressed lips to the inside of a small sealable transparent 17.7x18.8 cm polyethylene bag, simulating a light greeting kiss to the cheek), cough sample (2-3 light coughs at ~ 5-10 cm into an open sealable transparent 26.8x27.3 cm polyethylene bag) and speaking samples (continuous speech timed for a minimum of 3 but up to 5 minutes at 1-2 cm into an open sealable transparent 17.7x18.8 cm polyethylene bag) were collected at the bedside to which 2.5 ml, 5 ml and 3 ml, respectively, of DMEM+ was added. Hand samples with no specific instructions (obtained at the bedside shortly after room entry) were collected by washing each hand in 10 ml of DMEM+ for ~ 20-30 seconds in a sealable transparent 26.8x27.3 polyethylene bag. Facial tissue samples (discarded post nose blowing) were collected and placed in 8 ml of DMEM+. Cell phone samples, with no specific instructions, were collected with a 2 x 2 DMEM+ pre-moistened gauze, by wiping the glass surface and plastic back surface 3-4 times and then placing the gauze into 7 ml of DMEM+. The samples were then placed on ice or refrigerated within 1-2 hours at 4^0^ C and then stored at 4^0^ C for 12- 24 hours at the Public Health Laboratory within Foothills Medical Centre in Calgary ( a site within Alberta Precision Laboratories) and then transported to the Li Ka Shing Virology Institute BSL3 laboratories in Edmonton, Alberta, Canada with the authorization of the University of Alberta Human Research Ethics Board (Pro00099761). There, serial 10-fold dilutions of the virus were plated in duplicate on Vero cells (ATCC) and cultured for 3 days at 37˚C in Modified Eagle’s Medium (Gibco) supplemented with 10,000 units/ml of penicillin, 10,000 ug/ml of streptomycin and 25 ug/ml of Amphotericin B included (Gibco), 10% fetal bovine serum (Gibco), and 0.9% carboxymethylcellulose (Fisher). The cells were then fixed and stained with formaldehyde and crystal violet to permit plaque counts. E gene PCR for SARS-CoV-2 was performed as described by Berenger *et al* ^9^*.*  N1/N2 US CDC PCR (Integrated DNA Technologies; 2019-nCoV RUO Kit) was performed using a Qiagen RNA extraction kit, Promega GoTaq RT-qPCR reagents, and the Bio-Rad CFX96. Any additional samples submitted for diagnostic testing were done using Health Canada/FDA approved tests.
